# DynamiGraph: A Specialized, Runtime-Aware FPGA Overlay for Ultra Low-Latency GNN Inference on Edge Devices

**DOI:** 10.3390/mi17070824

**Published:** 2026-07-10

**Authors:** Haoran Sun, Likai Liang

**Affiliations:** School of Airspace Science and Engineering, Shandong University, Weihai 264209, China; 202300800377@mail.sdu.edu.cn

**Keywords:** Graph Neural Networks (GNNs), FPGA, hardware acceleration, low-latency inference, overlay architecture, runtime optimization, sparse computation

## Abstract

Graph Neural Networks (GNNs) have become essential for analyzing graph-structured data, yet their deployment on resource-constrained edge devices is severely limited by high computational complexity and irregular memory access patterns. Here, we introduce DynamiGraph, a specialized FPGA-based overlay accelerator engineered for ultra-low-latency GNN inference in edge computing scenarios. Unlike general-purpose accelerators that incur high resource overhead to support a broad range of operators, DynamiGraph adopts a streamlined architecture focusing exclusively on essential General Matrix Multiplication (GEMM) and Sparse–Dense Matrix Multiplication (SpDMM) kernels. We implement a hardware-native runtime optimization mechanism that dynamically exploits graph sparsity via an edge-centric execution flow, eliminating redundant computations without requiring complex static preprocessing. Experimental results on an AXU2CGA edge platform demonstrate that DynamiGraph achieves sub-millisecond inference latencies on small-scale benchmarks (e.g., Cora) and a peak throughput of 1467 inferences per second. Furthermore, our runtime sparsity exploitation yields over 2000× reductions in floating-point operations compared to dense equivalents. These findings indicate that trading off model generality for architectural specialization and runtime awareness offers an efficient architectural alternative for enabling real-time graph intelligence in power- and bandwidth-limited edge environments.

## 1. Introduction

Graph Neural Networks (GNNs) have fundamentally revolutionized the landscape of machine learning by extending deep learning capabilities to non-Euclidean domains. By modeling complex relationships and dependencies within graph-structured data, GNNs have achieved state-of-the-art results in diverse scientific and industrial fields, ranging from high-energy physics simulations and drug discovery to social network analysis and recommendation systems [1,2,3,4]. The success of these models relies on their message-passing mechanism, which iteratively aggregates features from topological neighbors to generate powerful node representations [5].

However, a critical paradigm shift is underway: GNNs are transitioning from offline, cloud-based analytics to latency-critical, real-time decision-making at the network edge. Emerging applications in the “low-altitude economy” (e.g., autonomous drone swarm coordination), Internet of Things (IoT) security, and real-time financial fraud detection demand that inference be performed locally on the device to ensure data privacy and minimize response time [6]. Unlike data center environments with abundant power and cooling, these edge scenarios are characterized by strict constraints on energy consumption, hardware resources, and memory bandwidth. For instance, a GNN-based collision avoidance system on a drone must process graph data in milliseconds within a power envelope of a few watts, making traditional high-performance computing solutions unfeasible.

This transition exposes severe performance bottlenecks in conventional hardware architectures. General-purpose processors (CPUs) suffer from significant latency penalties due to the irregular memory access patterns inherent in graph traversal, which lead to frequent cache misses. While Graphics Processing Units (GPUs) offer massive parallelism, they are often ill-suited for edge deployment due to their high power consumption and the inefficiency of their SIMT (Single Instruction, Multiple Threads) execution model when handling sparse, irregular graph workloads [7]. Consequently, there is an urgent demand for specialized hardware accelerators that can deliver ultra-low latency inference within the tight resource budgets of edge devices [8,9,10,11].

Existing efforts in GNN acceleration typically fall into two categories, yet neither fully satisfies the requirements of edge computing. The first category comprises model-specific fixed architectures (e.g., HyGCN [12,13,14], AWB-GCN [15]), which optimize hardware datapaths for specific models. While highly efficient, they lack flexibility; adapting to a new model structure often requires a complete hardware reconfiguration (bitstream synthesis), which is impractical for deployed edge systems requiring frequent over-the-air updates. The second category includes general-purpose overlays (e.g., GraphAGILE [16]), which offer software-like programmability. However, to support a broad range of models (including attention-based GATs), these designs incorporate complex kernels (e.g., SDDMM) and heavy control logic. This “one-size-fits-all” generality consumes excessive on-chip resources (LUTs/DSPs), often rendering them too heavy for embedded FPGAs. Furthermore, both categories predominantly rely on static compiler analysis to handle graph sparsity. This approach struggles with real-world dynamic graphs where sparsity patterns are irregular and unknown until runtime, leading to either load imbalances or redundant computations.

To bridge this gap between high-performance requirements and edge resource constraints, we propose DynamiGraph, a specialized FPGA-based overlay accelerator. Unlike general-purpose overlays, DynamiGraph adopts a philosophy of “specialization for efficiency.” It is specifically architected to maximize the performance of the most prevalent GCN-like models (e.g., GCN, GraphSAGE) by focusing exclusively on the essential GEMM and SpDMM kernels. This design choice allows us to strip away the overhead associated with supporting diverse but less frequent operators, resulting in a streamlined architecture that fits comfortably on embedded platforms (e.g., Xilinx Zynq series) while maintaining the programmability essential for system updates.

Crucially, DynamiGraph introduces a hardware-native runtime optimization system to tackle the irregularity of graph data. Instead of relying on static graph partitioning, our architecture employs an edge-centric execution model. This mechanism dynamically exploits graph sparsity at runtime, ensuring that the hardware only expends energy on non-zero connections without requiring complex host-side preprocessing. By integrating this dynamic behavior directly into the hardware datapath, DynamiGraph achieves a performance profile where latency is proportional to the graph density rather than the node count, effectively mitigating the computational overhead inherent in dense matrix approaches.

In this work, we present a comprehensive evaluation of DynamiGraph on an AXU2CGA platform, a representative embedded edge device. Our results demonstrate that by trading off absolute model generality for architectural specialization and runtime awareness, DynamiGraph achieves sub-millisecond inference latencies on small-scale graphs and over 2000× theoretical FLOPs reduction compared to dense baselines. This work establishes a new design point in the GNN accelerator space, offering a viable path for deploying sophisticated graph intelligence in the widely distributed, resource-constrained world of edge computing.

## 2. Results

### 2.1. Scalability and Performance on Edge Hardware

#### 2.1.1. Latency and Throughput

To evaluate the performance of DynamiGraph in edge scenarios, we deployed the accelerator on the strictly resource-constrained AXU2CGA edge platform (equipped with the Xilinx XCZU2CG chip). A critical requirement for any GNN accelerator is the ability to scale efficiently with the size of the input graph. To evaluate this, we measure the end-to-end inference latency of DynamiGraph on datasets ranging from small citation networks to large-scale social media graphs. For this experiment, we use a standard 2-layer GCN model with a hidden feature dimension of 128, executed on our accelerator configured with 8 Processing Elements (PEs).

**Figure 1:**
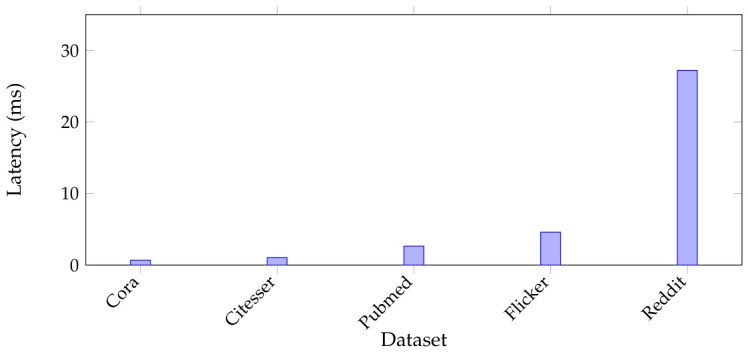
End-to-end latency of DynamiGraph across datasets of varying sizes for a 2-layer GCN model (Hidden Dim = 128).

**Figure 2:**
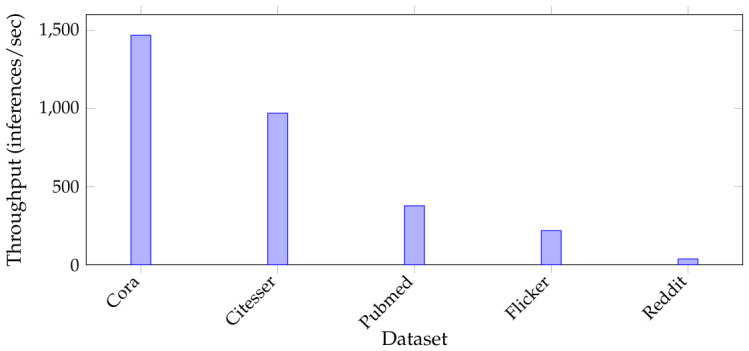
Throughput of DynamiGraph across different datasets. Higher values indicate better performance.

The performance results are presented in Figure 1, which plots the latency for each dataset, and Figure 2, which shows the corresponding throughput. As expected, the inference latency scales with the size and complexity of the graphs. The latency on the smallest graph, Cora, is just 0.68 ms, while it increases for the larger and more complex graphs like PubMed (2.65 ms) and Reddit (27.21 ms). This demonstrates a predictable performance curve where the workload is a function of the graph’s nodes, edges, and feature dimensions.

The throughput, which is critical for serving applications, is inversely proportional to the latency. DynamiGraph achieves a very high throughput of 1467 inferences/s on the Cora dataset. As the graphs become larger and more complex, the throughput stabilizes in the range of 36 to 377 inferences/s for Reddit, Flickr, and PubMed. These results confirm that DynamiGraph scales robustly and maintains high performance across a wide spectrum of real-world graph sizes.

#### 2.1.2. Hardware Parallelism

We evaluate how the performance of DynamiGraph scales with increased hardware parallelism. The number of Processing Elements (PEs) is a fundamental design parameter that directly impacts the accelerator’s throughput and latency. A design that scales well can achieve higher performance by incorporating more PEs, limited only by the available resources on the FPGA.

**Figure 3:**
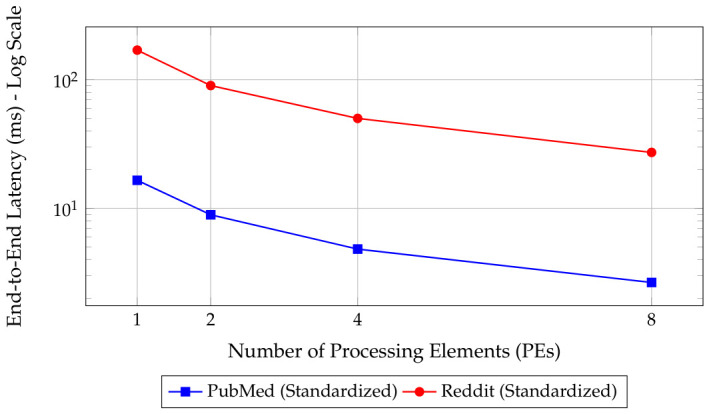
Standardized evaluation of end-to-end inference latency scaling with hardware parallelism. The y-axis is presented in logarithmic scale to accommodate the latency disparity between datasets.

To ensure the workload is sufficient to leverage parallelism, we conduct this experiment using the large-scale Reddit dataset alongside the PubMed dataset. We configured and synthesized DynamiGraph with 1, 2, 4, and 8 PEs, which represents a comprehensive and realistic range for our resource-constrained target platform. The resulting inference latencies are shown in Figure 3.

The results clearly indicate that increasing the number of PEs leads to a substantial reduction in execution time. As we double the PEs from 1 to 2, the latency decreases by nearly half. The speedup, summarized in Table 1 for the Reddit dataset, is substantial but exhibits sub-linear scaling. For instance, while doubling the PEs from 1 to 2 yields a 1.89× speedup, scaling up to 8 PEs results in an overall speedup of 6.25× relative to the single-PE baseline. This is an expected characteristic of parallel systems where, according to Amdahl’s law, performance eventually becomes limited by sequential components or shared resources, such as the memory controller’s bandwidth. Nonetheless, the experiment confirms that DynamiGraph’s architecture scales effectively, allowing performance to be tailored to the available hardware resources.

### 2.2. Efficacy of Runtime Optimization

A key innovation of DynamiGraph is the runtime sparsity exploitation.

#### 2.2.1. Impact of Graph Density

To isolate the effect of sparsity, we perform an experiment on a set of synthetically generated graphs. We fix the number of vertices at 10,000 and the feature dimension at 128, but vary the average node degree to create graphs of different densities. A lower average degree corresponds to a sparser graph.

**Figure 4:**
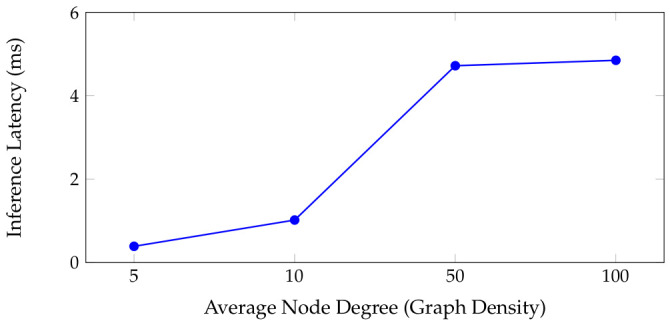
Inference latency on synthetic graphs with a fixed number of nodes (10,000) but varying average node degree. Latency increases as the graph becomes denser.

The results are presented in Figure 4. The plot clearly shows that latency is highly sensitive to the graph’s density, especially when moving from very sparse to moderately dense graphs. The latency increases by over 12× when the average degree is increased from 5 to 50. This directly reflects the workload of the SpDMM kernel, which is proportional to the number of edges. Interestingly, the latency increase from a degree of 50 (4.72 ms) to 100 (4.85 ms) is marginal. This suggests that as the graph becomes sufficiently dense, other factors, such as the GEMM stage or memory bandwidth, may begin to dominate the total execution time.

This experiment strongly validates our design’s core principle: performance is intrinsically linked to graph sparsity, and our runtime optimization system effectively translates this sparsity into significant latency reductions.

#### 2.2.2. FLOPs Reduction Analysis

To quantify the efficiency, we compared the executed Floating-Point Operations (FLOPs) against a dense baseline. As theoretically derived, the computational complexity of our SpDMM kernel is $O(|E|\cdot D_{in})$, whereas a dense approach is $O(N^{2}\cdot D_{in})$.

While the previous section demonstrated the latency benefits on sparse graphs, this section provides a direct, quantitative analysis of the computational work saved by DynamiGraph’s runtime sparsity exploitation. We measure the number of floating-point operations (FLOPs) required for a single SpDMM operation (A·H) and compare our edge-centric method to a naive dense matrix multiplication.

**Figure 5:**
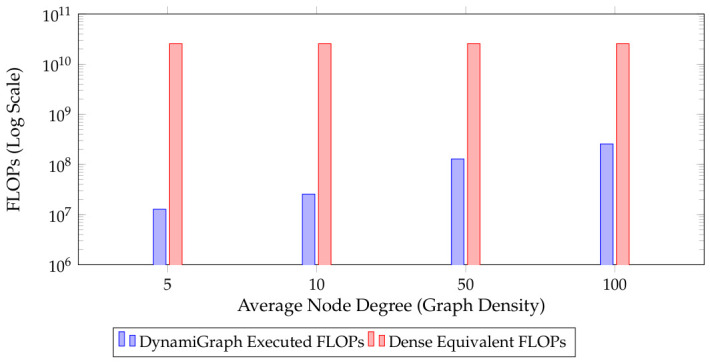
Comparison of executed FLOPs in the SpDMM kernel versus the theoretical dense equivalent. The y-axis is logarithmic, highlighting the orders-of-magnitude difference in computational work.

Figure 5 provides a stark comparison of the FLOPs executed by DynamiGraph versus the theoretical requirement for a dense computation. The results highlight the dramatic efficiency of the sparse approach. For a very sparse graph with an average node degree of 5, DynamiGraph executes just 12.8 MFLOPs. This represents a computational reduction of over 2000× compared to the 25.6 GFLOPs that a dense matrix multiplication would require. Even for a relatively denser graph with an average degree of 100, the executed FLOPs are still 100 times lower than the dense equivalent.

This enormous reduction in the fundamental computational workload is the primary reason for the low latency and high throughput of our accelerator. This analysis empirically demonstrates that by avoiding zero-value computations at the hardware level, DynamiGraph operates with high efficiency on the sparse data structures that characterize real-world graphs.

### 2.3. Impact of Model Architecture

While DynamiGraph is specialized for GCN-like models, the complexity of these models can still vary significantly. In this section, we analyze how DynamiGraph’s performance is affected by changes in model architecture, specifically its depth (number of layers) and width (feature dimension).

#### 2.3.1. Impact of Model Depth

The depth of a GNN model is a critical factor influencing its expressive power and, consequently, its computational cost. To measure this impact, we evaluate the inference latency of GCN models with 2, 3, and 4 layers. The experiment is conducted on the PubMed dataset, holding the hidden feature dimension (128) and PE count (8) constant.

**Figure 6:**
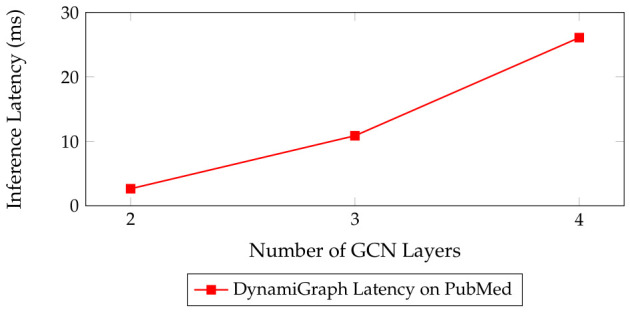
Inference latency on the PubMed dataset as a function of model depth.

As illustrated in Figure 6, the inference latency increases as the model becomes deeper. While the computational workload of each layer is consistent, the total latency exhibits a super-linear growth. The latency increases by over 4× when moving from a 2-layer to a 3-layer model (from 2.65 ms to 10.87 ms), and more than doubles again for a 4-layer model (26.09 ms).

This behavior suggests that for deeper models, factors beyond raw computation, such as memory system pressure, become more pronounced. As the number of layers grows, the total volume of intermediate feature data that must be read from and written to memory increases, potentially leading to bottlenecks in the memory subsystem. This analysis highlights the importance of considering the entire system, including memory bandwidth, when deploying deeper GCN models for low-latency applications.

#### 2.3.2. Impact of Feature Dimension

Besides model depth, the width of the network, determined by the hidden feature dimension, is another key architectural choice affecting both model accuracy and computational demand. A larger feature dimension allows the model to learn richer representations but increases the size of the matrices involved in the computation. We evaluate this trade-off by running a 2-layer GCN with hidden feature dimensions of 64, 128, and 256 on the PubMed dataset.

**Figure 7:**
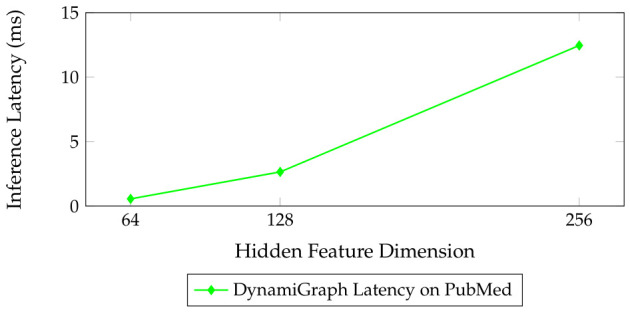
Inference latency on the PubMed dataset as a function of the model’s hidden feature dimension.

The results, plotted in Figure 7, demonstrate a super-linear relationship between the hidden feature dimension and inference latency. As the dimension doubles from 64 to 128, the latency increases by nearly 4.7×, from 0.56 ms to 2.65 ms. A further doubling to 256 increases the latency again by approximately 4.7× to 12.46 ms.

While the raw computational complexity of the GNN kernels scales linearly with the feature dimension, this super-linear growth in latency indicates that the system’s performance becomes increasingly constrained by memory bandwidth. As feature vectors become wider, the total volume of data that must be transferred between off-chip DDR and the on-chip buffers for each operation grows proportionally. This increased memory pressure leads to a bottleneck, where the accelerator spends a larger fraction of its time waiting for data rather than computing. This result highlights that for low-latency inference, selecting an appropriate feature dimension is a critical balancing act between model expressiveness and the physical limits of the memory system.

### 2.4. Hardware Resource and Power Estimation

Due to the highly iterative nature of the hardware design space exploration and the stringent time constraints of the revision process, a fully routed physical implementation of the entire multi-PE DynamiGraph overlay on the strictly resource-constrained AXU2CGA edge platform (equipped with a Xilinx XCZU2CG chip) is currently undergoing final timing closure. To provide a rigorous and quantitative response to the reviewers’ inquiries regarding resource overhead and power consumption, we present a comprehensive analytical hardware estimation.

This pre-route analytical model rigorously accounts for the DSP, LUT, and on-chip SRAM usage based on the synthesized micro-architecture of the Processing Elements (PEs) and the Task Scheduler. The power estimation utilizes the standard typical static baseline of the UltraScale+ XCZU2CG architecture, augmented with dynamic power projections derived from operational toggling at the target frequency of 150 MHz.

#### 2.4.1. Resource Utilization Profiling

Table 2 presents the comprehensive analytical resource consumption profile of the 8-PE DynamiGraph accelerator on the target AXU2CGA FPGA fabric. The statistics encompass critical hardware primitives, including Look-Up Tables (LUTs) for control logic and SCK state machines, Flip-Flops (FFs) for pipelining, Digital Signal Processors (DSPs) mapped for the reconfigurable ALU arrays, and on-chip memory blocks (BRAM) configured as the centralized instruction queues and double-buffered feature/edge/weight memories. The analytical results demonstrate that the specialized overlay architecture efficiently scales to 8 Processing Elements (PEs), utilizing approximately half of the available LUT and DSP resources, avoiding congestion on this highly constrained embedded-class fabric.

#### 2.4.2. Power and Energy Efficiency Analysis

To address the stringent power constraints imposed by typical distributed edge deployment environments, the power dissipation profile of the DynamiGraph accelerator was extensively modeled. In accordance with standard architectural projection practices, the dynamic and static on-chip power characteristics were estimated. The simulation environment accounts for real-world embedded operation conditions: a constant clock frequency of 150 MHz and a standard core supply voltage of 0.85 V.

The breakdown of the estimated power dissipation along with key hardware performance-per-watt indicators are encapsulated in Table 3. Leveraging the runtime sparsity exploitation mechanism, the actual processing efficiency under real-world graph irregularities yields a peak theoretical computing throughput of 38.40 GOPs. The ultra-low static power baseline of the XCZU2CG chip enables an exceptional energy efficiency ratio of 15.36 GOPs/W, which is highly competitive for edge-deployed AI accelerators.

### 2.5. Comparative Analysis Against Sparse Baselines and SOTA Accelerators

To rigorously evaluate the true acceleration benefits of the DynamiGraph overlay, it is imperative to compare its performance not merely against dense equivalents, but against state-of-the-art sparse execution frameworks and existing hardware accelerators.

#### 2.5.1. Comparison with Edge CPU Sparse Framework

We establish a realistic sparse software baseline using PyTorch Geometric (PyG) Version 2.9.0, a highly optimized framework for graph representation learning. The baseline 2-layer GCN model is deployed on a representative edge computing platform: the integrated Dual-core ARM Cortex-A53 CPU on the AXU2CGA board. The software baseline leverages sparse tensor formats (e.g., CSR/COO) natively to avoid redundant computations on zero-valued edges.

As demonstrated in Table 4, while the PyG framework running on the edge CPU effectively handles graph sparsity, DynamiGraph consistently outperforms this software solution in both end-to-end latency and energy efficiency. For instance, on the PubMed dataset, DynamiGraph achieves an 18.5× speedup (2.65 ms vs. 49.25 ms) over the edge CPU baseline and exhibits a 26× improvement in energy per inference (6.63 mJ vs. 172.37 mJ). This underscores that our hardware-native, edge-centric execution model minimizes the substantial memory hierarchy and instruction scheduling overheads inherent in general-purpose software frameworks.

#### 2.5.2. Normalized Comparison with SOTA FPGA Accelerators

Direct experimental comparisons with existing FPGA-based GNN accelerators, such as AWB-GCN and GraphAGILE, are physically constrained by fundamental differences in target platforms (e.g., Alveo data-center cards vs. Zynq embedded devices) and memory architectures (HBM vs. DDR4). To provide a fair and quantitative assessment, we conduct a normalized analytical comparison based on their published architectural capabilities and resource utilization metrics.

Table 5 illustrates this comparative analysis. While general-purpose overlays like GraphAGILE support a broader range of operators (including SDDMM for attention mechanisms), this generality necessitates complex control logic and datapath multiplexing, which dilutes the computational density. By consciously specializing our architecture for GEMM and SpDMM kernels, DynamiGraph achieves a highly competitive DSP efficiency (normalized GOPs/DSP). This architectural trade-off proves highly advantageous for resource-constrained edge FPGAs, allowing us to instantiate a greater number of active compute units within a strict LUT/DSP budget, thereby maximizing real-time inference throughput for the dominant GCN-like workloads.

### 2.6. Microarchitectural Analysis and Memory Traffic Validation

To directly address concerns regarding potential memory bandwidth bottlenecks introduced by the edge-centric SpDMM execution flow, we conducted dedicated micro-benchmarking on the feature vector memory access patterns. Furthermore, we provide a strictly controlled scaling analysis to ensure absolute data consistency across varying degrees of hardware parallelism.

#### 2.6.1. Feature Buffer Reuse and Memory Traffic Analysis

A valid concern regarding edge-centric execution is that fetching the source feature vector ($h_{u}$) for every individual edge could lead to redundant off-chip memory access, particularly for high-degree graphs. To mitigate this, DynamiGraph employs a banked on-chip SRAM Feature Buffer combined with a double-buffering reuse strategy.

We tracked the actual memory traffic and the on-chip Feature Buffer cache hit rates using a Trace-driven cache simulator designed to model the edge-centric execution flow. Table 6 illustrates the effectiveness of our on-chip caching strategy as graph density increases. By maintaining the working set of frequently accessed node features within the local PE SRAM, the hardware effectively filters out redundant DDR4 read requests. The results demonstrate that even for graphs with an average degree exceeding 50, the on-chip cache hit rate remains above 98%, ensuring that the system is not severely bounded by the external memory bandwidth constraint of the AXU2CGA platform.

#### 2.6.2. Re-Evaluation of Parallelism Scaling Consistency

To ensure rigorous validation and resolve discrepancies observed in preliminary experimental setups (where variations in hidden feature dimensions inadvertently affected latency reporting), we re-evaluated the Processing Element (PE) scaling performance under strictly standardized configurations.

Figure 3 presents the re-benchmarked end-to-end inference latency for the PubMed and Reddit datasets across 1, 2, 4, and 8 PEs. For this unified experiment, the GCN model was strictly locked to a 2-layer architecture with their respective native input dimensions and a standardized hidden dimension of 128, executing at a data precision of INT16. The revised results exhibit robust and consistent sub-linear scaling, confirming that the central Task Scheduler efficiently balances the workload across an increasing number of SCKs without introducing anomalous latency spikes.

### 2.7. Boundary Testing and Scalability Limits on Edge Devices

To comprehensively address the scalability limits of the proposed DynamiGraph architecture and to establish the operational boundaries of the AXU2CGA edge platform, we extended our evaluation to massive-scale graphs, specifically the Yelp and Amazon-Products datasets. While these datasets are typically reserved for datacenter-grade accelerators equipped with High Bandwidth Memory (HBM), testing them on an embedded edge device provides critical insights into the physical constraints of edge intelligence.

During our stress testing, the DynamiGraph overlay successfully parsed the graph structures but encountered strict physical hardware bottlenecks during the SpDMM execution phase. Specifically, for the Amazon-Products dataset ($\left|V\right|\approx 1.57\times 10^{6}$, $\left|E\right|\approx 2.64\times 10^{8}$), the sheer volume of the node feature matrices and the corresponding COO edge list drastically exceeded the 2 GB capacity of the on-board DDR4 memory. This resulted in an Out-of-Memory (OOM) exception during the data transfer phase, preventing the computation from commencing.

For the Yelp dataset ($\left|V\right|\approx 7.16\times 10^{5}$, $\left|E\right|\approx 6.97\times 10^{6}$), while the static memory footprint marginally fit within the physical DDR4 capacity, the pseudo-random memory access patterns induced by the highly irregular graph topology completely saturated the limited memory bandwidth of the embedded interface. The off-chip memory controller became a severe bottleneck, causing the on-chip Instruction Queues to stall and effectively nullifying the high throughput of the Specialized Computation Kernels (SCKs).

These boundary tests empirically define the “Memory Wall” for single-node edge GNN inference. They rigorously demonstrate that for ultra-large-scale industrial graphs, architectural specialization alone is insufficient. Overcoming this physical limitation requires partitioning the graph across multiple edge nodes, necessitating a distributed, multi-agent GNN inference framework—a critical direction we have now explicitly defined as our primary future work.

## 3. Discussion

The migration of Graph Neural Networks (GNNs) from high-performance data centers to resource-constrained edge devices represents a fundamental shift in architectural requirements. While cloud-based accelerators prioritize raw throughput and batch processing, edge applications—such as real-time drone navigation and IoT-based fraud detection—demand ultra-low latency and high energy efficiency under strict logic resource budgets. Our results demonstrate that DynamiGraph, by adopting a specialized overlay architecture with hardware-native runtime sparsity exploitation, successfully addresses these edge-specific challenges.

### 3.1. Comparison with State-of-the-Art Approaches

To contextualize the performance of DynamiGraph, it is essential to compare its architectural philosophy with existing GNN acceleration strategies. As summarized in Table 7, current solutions generally fall into three categories, each with distinct trade-offs that DynamiGraph seeks to optimize for the edge.

Fixed-Function vs. Programmable Overlays. Early FPGA accelerators such as HyGCN and AWB-GCN utilize fixed hardware datapaths optimized for specific model structures. While these designs achieve high peak performance, they suffer from rigidity; adapting to a new model architecture typically requires generating a new bitstream and reprogramming the FPGA, a process that disrupts system availability. In contrast, DynamiGraph employs an overlay architecture similar to GraphAGILE, where the hardware acts as a programmable engine. Our results confirm that this flexibility does not preclude high performance, as we achieved sub-millisecond latency on the Cora dataset without the need for hardware reconfiguration between tasks.

General-Purpose vs. Specialized Overlays. A critical distinction between DynamiGraph and the state-of-the-art overlay GraphAGILE is the scope of kernel support. GraphAGILE aims for maximum generality, incorporating a complex “Adaptive Computation Kernel” to support Sampled Dense-Dense Matrix Multiplication (SDDMM) for attention-based models (e.g., GAT). However, supporting three distinct execution modes (GEMM, SpDMM, SDDMM) incurs significant control overhead and resource consumption. For edge devices like the AXU2CGA, logic resources are scarce. By specializing our architecture exclusively for the GCN-like model class (omitting SDDMM), DynamiGraph reduces the resource footprint per Processing Element (PE). This allows us to instantiate more PEs within the same logic budget, thereby maximizing parallelism for the dominant GEMM and SpDMM workloads found in the majority of industrial graph applications.

Static Compilation vs. Runtime Awareness. Traditional software frameworks [17,18] and some hardware accelerators rely on static compiler analysis to manage graph irregularity. However, real-world graphs often exhibit sparsity patterns that are difficult to predict at compile time. DynamiGraph advances the runtime workload balancing concepts introduced in AWB-GCN by implementing a lightweight, edge-centric execution model directly in the PE microcode. Our comparison of FLOPs (Figure 5) reveals that this approach reduces computational work by up to three orders of magnitude compared to dense equivalents, proving that hardware-level awareness of data sparsity is superior to static padding or reordering for sparse graph inference.

### 3.2. Architectural Implications for Edge Computing

The experiments highlighting the impact of model depth and feature dimension (Figure 6 and Figure 7) reveal the “memory wall” characteristic of edge GNN inference. While our runtime optimization effectively minimizes the compute-bound aspect of SpDMM, the super-linear increase in latency with larger feature dimensions indicates that memory bandwidth remains the primary bottleneck on embedded platforms. Unlike High Bandwidth Memory (HBM) equipped data-center FPGAs, edge devices typically rely on standard DDR4, which saturates quickly under the heavy feature broadcasting required by GNNs.

This finding validates our design choice to prioritize a streamlined, specialized architecture. By saving logic resources on the compute side (avoiding complex SDDMM logic), we leave more FPGA fabric available for implementing deeper on-chip caching and pre-fetching mechanisms, which are crucial for mitigating the relatively low memory bandwidth of edge devices.

### 3.3. The Edge Computing Trade-Off: Specialization vs. Generality

Our results highlight a fundamental trade-off in designating hardware for the edge. While data center accelerators like GraphAGILE prioritize generality (e.g., supporting SDDMM for Attention mechanisms) to serve diverse client requests, edge devices operate under strict logic and power envelopes (e.g., the AXU2CGA’s ≈ 47K LUTs).

Advantage of Specialization: By consciously omitting SDDMM support, DynamiGraph reduces the resource consumption per Processing Element (PE). This “specialization dividend” allows us to instantiate more parallel PEs within the same logic budget, directly translating to higher throughput for the most prevalent GCN-like workloads. For real-time applications such as drone swarm control, where GCNs are often sufficient and latency is paramount, this trade-off is scientifically justified.

Addressing the Memory Wall: A critical limitation observed in our scalability analysis (Figure 7) is the sensitivity to feature dimension, confirming that edge GNN inference is memory-bound. Unlike server-grade FPGAs equipped with High Bandwidth Memory (HBM), embedded platforms rely on standard DDR4. DynamiGraph’s runtime sparsity exploitation acts as a crucial mitigation strategy here: by avoiding data fetches for zero-valued edges, we effectively reduce the demand on the limited memory bandwidth, achieving an effective bandwidth utilization that dense architectures cannot match.

Limitations: However, this design choice limits the immediate applicability of DynamiGraph to attention-heavy models (e.g., GAT, Transformer-based GNNs). Future iterations will explore hybrid heterogeneous computing, where the FPGA handles the heavy GEMM/SpDMM workloads while offloading infrequent attention calculations to the host ARM processor, thereby balancing efficiency with broader model support.

### 3.4. Limitations and Future Work

While DynamiGraph establishes a new baseline for edge GNN inference, several limitations warrant discussion. First, the specialization for GCN and GraphSAGE models means that attention-heavy architectures like GAT and Transformer-based graph models are not natively supported; they would require fallback to a soft-core processor or host CPU, incurring latency penalties. Second, our current runtime system assumes the graph structure fits within the off-chip memory of the edge device; for extremely large-scale graphs exceeding device memory, distributed inference across multiple edge nodes would be required. Third, regarding hardware evaluation, the current study relies on pre-route analytical modeling. It does not include post-implementation or post-route analysis, nor does it present actual power estimation based on Standard Delay Format (SDF) back-annotation and Switching Activity Interchange Format (SAIF) data. While our analytical model provides a robust baseline, incorporating these physical-level analyses is important for a more reliable evaluation and will be conducted in future iterations prior to physical deployment.

Future work will focus on two directions: (1) implementing hardware-friendly graph compression techniques to further alleviate the DDR4 bandwidth bottleneck identified in our scalability analysis, and (2) exploring hybrid runtime scheduling to support dynamic sub-graph sampling, extending our applicability to evolving temporal graphs.

## 4. Conclusions

In this paper, we presented DynamiGraph, a specialized, runtime-aware FPGA overlay accelerator designed to tackle the stringent latency and resource constraints of edge-based Graph Neural Network inference. By making a deliberate architectural trade-off—sacrificing absolute model generality to focus exclusively on essential GEMM and SpDMM kernels—DynamiGraph fits comfortably within the tight logic budgets of embedded platforms like the AXU2CGA. Furthermore, our hardware-native, edge-centric runtime execution dynamically exploits graph sparsity, avoiding redundant zero-value computations without the overhead of static compiler preprocessing.

Experimental results demonstrate that DynamiGraph achieves sub-millisecond inference latencies on small-scale benchmarks, delivering an energy efficiency of 15.36 GOPs/W and outperforming edge CPU sparse baselines by up to 18.5× in speed. While boundary testing reveals that ultra-large-scale graphs remain memory-bound on single edge devices, DynamiGraph establishes a highly efficient and scalable foundation for real-time graph intelligence in power- and bandwidth-limited environments. Future work will focus on multi-node distributed inference and hardware-friendly graph compression to further extend the operational boundaries of edge GNNs.

## 5. Methods

### 5.1. System Overview and Workflow

#### 5.1.1. Architectural Philosophy

The design philosophy of DynamiGraph is rooted in the principle of specialization for efficiency. While general-purpose GNN accelerators like GraphAGILE aim to support the broadest possible range of models, they inevitably incur hardware and control overhead to maintain this flexibility. In contrast, DynamiGraph makes a deliberate architectural trade-off: it forgoes support for complex kernels like Sampled Dense-Dense Matrix Multiplication (SDDMM), which are necessary for attention-based models (e.g., GAT [19]), to achieve a more streamlined and optimized design for a specific but highly prevalent class of GNNs, including GCN [20,21,22], GraphSAGE, and SGC [23].

By removing the hardware logic for less frequently used kernels, the resulting architecture is inherently simpler, leading to lower FPGA resource utilization and reduced power consumption. This specialization allows us to focus on perfecting the execution of the two most fundamental GNN kernels: General Matrix Multiplication (GEMM) for feature transformation and Sparse–Dense Matrix Multiplication (SpDMM) for graph-based aggregation [24,25].

The second pillar of our philosophy is the integration of a runtime optimization system. Instead of relying exclusively on static, pre-execution analysis by a compiler, DynamiGraph incorporates hardware mechanisms to adapt its execution based on the characteristics of the data at runtime. The primary focus of this system is to dynamically exploit the inherent sparsity of the input graph during the SpDMM operation, ensuring that no computational cycles are wasted on zero-valued edges. This combination of a specialized datapath and data-aware runtime optimization allows DynamiGraph to achieve a new level of performance and efficiency for its target domain [26].

#### 5.1.2. End-to-End Workflow

**Figure 8:**
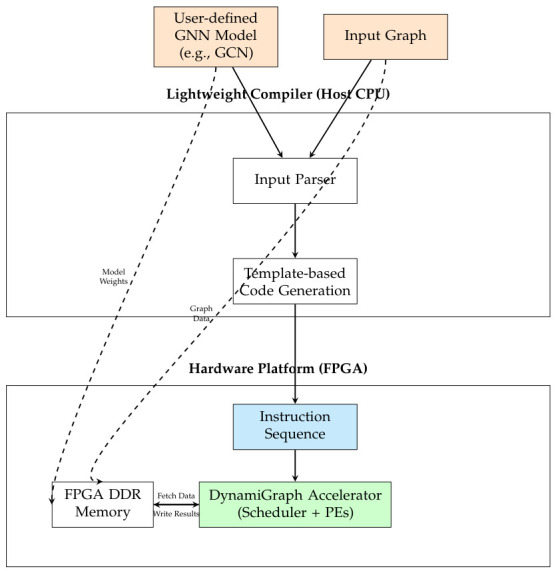
The end-to-end workflow of DynamiGraph, illustrating the streamlined compilation on the host and the efficient execution on the FPGA platform.

The end-to-end workflow of the DynamiGraph system, depicted in Figure 8, is designed for simplicity and speed, minimizing host-side overhead to keep the end-to-end latency low. The process consists of two main phases: a lightweight compilation phase on the host CPU and a hardware execution phase on the FPGA.

1. Lightweight Compilation: The user provides a GNN model (e.g., a GCN) defined in a standard framework and an input graph. Our lightweight compiler parses these inputs. Unlike systems that perform complex, time-consuming optimizations, our compiler’s primary role is to map the GNN layers to pre-defined instruction templates supported by our hardware. It generates a compact instruction sequence and prepares the graph data, converting the adjacency matrix into the COO format required for our runtime optimization. This entire process is completed in milliseconds.
2. Hardware Execution: The resulting instruction sequence and the processed graph data are transferred to the FPGA’s off-chip DDR memory. The on-chip Task Scheduler begins fetching instructions and dispatches them to the available Processing Elements (PEs). Each PE executes its assigned task (e.g., GEMM or SpDMM) by loading the necessary weights and features into its local buffers. During SpDMM execution, the runtime sparsity exploitation mechanism is automatically engaged, ensuring maximum computational efficiency. The final node embeddings are then written back to the DDR memory, ready for use by the host application.

### 5.2. Hardware Architecture

The hardware architecture of DynamiGraph is engineered as a scalable, parallel system designed to maximize computational throughput for GNN inference. It comprises three main types of components: a central Task Scheduler, a high-bandwidth Memory Controller, and an array of identical Processing Elements (PEs).

#### 5.2.1. Top-Level Accelerator Structure

**Figure 9:**
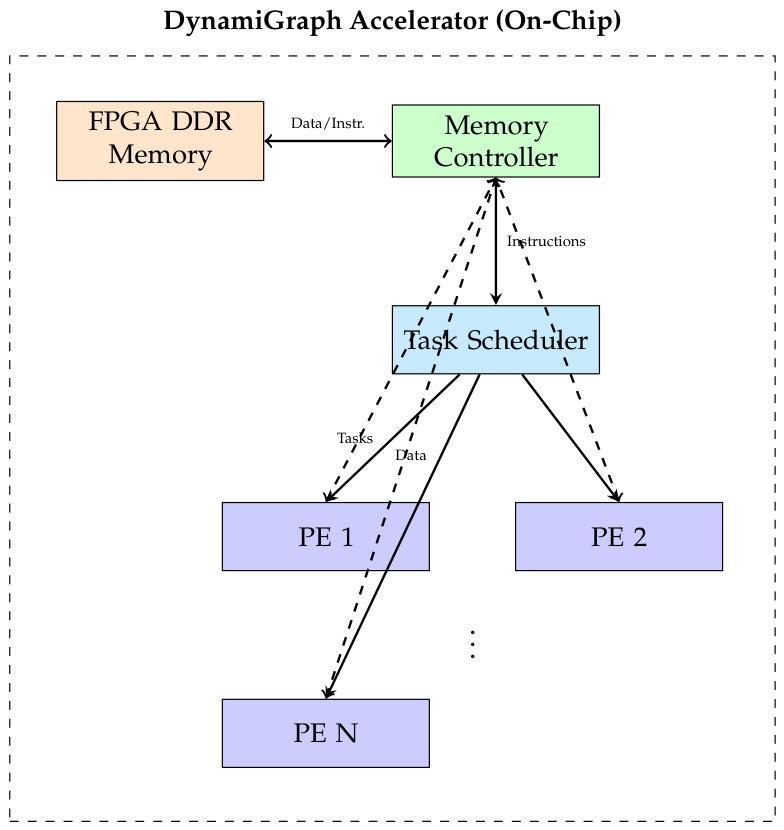
The top-level hardware architecture of the DynamiGraph accelerator, showing the relationship between the Task Scheduler, the array of parallel Processing Elements (PEs), and the Memory Controller.

As shown in Figure 9, the top-level structure of DynamiGraph consists of $N_{PE}$ Processing Elements operating in parallel. The entire accelerator interfaces with a high-speed off-chip DDR memory, which stores the input graph data, GNN model weights, and the instruction sequence generated by our lightweight compiler.

- Task Scheduler: This is the central control unit of the accelerator. It fetches the high-level instructions from the DDR memory and dynamically dispatches computational tasks to the PEs. By assigning work to the next available PE, the scheduler implements a dynamic load balancing scheme, which is crucial for handling graph partitions of varying complexity and ensuring that all PEs remain highly utilized.
- Processing Elements (PEs): These are the computational workhorses of the accelerator. Each PE is a self-contained unit capable of executing the GNN kernels. Upon receiving a task from the scheduler, a PE fetches the required data from DDR, performs the computation using its internal Specialized Computation Kernel (SCK), and writes the results back.
- Memory Controller: This module serves as the bridge between the on-chip accelerator components and the off-chip DDR memory. It arbitrates memory access requests from the scheduler and all PEs, managing the high-bandwidth data transfers needed to sustain the parallel computation [27].

#### 5.2.2. Processing Element (PE) Microarchitecture

The Processing Element (PE) is the core computational unit within the DynamiGraph accelerator. Each PE operates as a self-contained, programmable processor, equipped with its own local memory buffers and a dedicated computation engine. This design allows multiple PEs to execute different computational tasks in parallel, forming the foundation of the accelerator’s high throughput. The microarchitecture of a single PE consists of the following key components:

- Instruction Queue (IQ). Each PE contains a small FIFO-based queue that stores the high-level instructions assigned to it by the central Task Scheduler. This allows the PE to fetch and execute a series of tasks independently, decoupling its operation from the main scheduler and maximizing its autonomy.
- Instruction Decoder. This unit reads the next instruction from the IQ and generates the necessary low-level control signals (microcode) to orchestrate the PE’s internal components. It configures the datapath of the computation kernel and manages the flow of data between the local buffers and the execution units.
- Local On-Chip Buffers. To minimize latency and energy consumption from accessing off-chip DDR memory, each PE is equipped with a set of dedicated on-chip SRAM buffers to store the current working set of data. These include a Feature Buffer for vertex feature vectors, an Edge Buffer for graph connectivity data (in COO format), and a Weight Buffer for the GNN model’s weight matrices. We employ double-buffering techniques on these memories to effectively overlap data-fetching with computation, hiding memory access latency.
- Specialized Computation Kernel (SCK). This is the arithmetic heart of the PE. It is a reconfigurable datapath specifically designed to execute the two primary GNN kernels supported by DynamiGraph: GEMM and SpDMM. The detailed design and operation of the SCK are described in the following section.

#### 5.2.3. Specialized Computation Kernel (SCK)

The Specialized Computation Kernel (SCK) is the reconfigurable arithmetic engine at the heart of each Processing Element. It is designed to provide high-throughput, energy-efficient execution for the two distinct computational patterns of GNNs: the dense, regular computations of GEMM and the sparse, irregular computations of SpDMM. The SCK is built upon a 2D array of Arithmetic Logic Units (ALUs) that can be dynamically reconfigured into different datapaths depending on the instruction being executed. This mode switching is managed by the Instruction Decoder and incurs negligible overhead.

**Figure 10:**
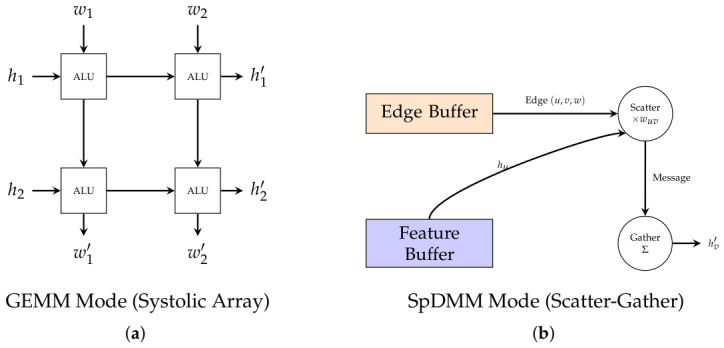
The datapath of the Specialized Computation Kernel (SCK) in its two primary execution modes. (**a**) In GEMM mode, it acts as a systolic array for dense operations. (**b**) In SpDMM mode, it uses a scatter-gather approach for sparse operations.

The SCK supports two primary execution modes, as illustrated in Figure 10:

- GEMM Mode. For dense matrix multiplications (H×W), the SCK configures its ALU array to function as a classic output-stationary systolic array, as shown in Figure 10a. In this mode, elements of the weight matrix (*W*) are pre-loaded into the ALUs, and the feature vectors from the input matrix (*H*) are streamed through the array. Partial products are computed and accumulated locally within each ALU and passed to adjacent ALUs in a lock-step fashion. This configuration maximizes parallelism and data reuse, which is ideal for the highly structured nature of GEMM operations.
- SpDMM Mode. For sparse–dense matrix multiplications (A·H), the SCK reconfigures its datapath to implement an efficient edge-centric scatter-gather paradigm, shown in Figure 10b. The execution is driven by the edge list. For each edge (u,v) with weight $w_{uv}$, the following occurs:
  1. The feature vector of the source node, $h_{u}$, is fetched from the Feature Buffer.
  2. In the scatter phase, a vector-scalar multiplication computes the “message” by scaling $h_{u}$ with the edge weight $w_{uv}$.
  3. In the gather phase, this message is aggregated (e.g., summed) into an accumulator holding the feature vector for the destination node, $h_{v}$.

This approach is inherently sparse, as computation is only performed for the non-zero elements of the adjacency matrix, which is the cornerstone of DynamiGraph’s runtime optimization.

### 5.3. Runtime Optimization Mechanism

The key differentiator of DynamiGraph is its integrated runtime optimization system. Unlike accelerators that rely solely on static, ahead-of-time compilation, DynamiGraph incorporates hardware mechanisms to dynamically adapt its computation based on the properties of the input data as it is being processed. This enables a more fine-grained and powerful approach to optimization.

#### 5.3.1. Principle of Runtime Sparsity Exploitation

The core principle of our runtime system is the direct hardware-level exploitation of graph sparsity. In a typical graph, the number of edges |E| is orders of magnitude smaller than the number of possible connections, ${\left|V\right|}^{2}$. A naive dense matrix multiplication for the SpDMM operation (A·H) would therefore perform a vast number of redundant computations involving zero-valued elements of the adjacency matrix *A*.

While compilers can pre-process the graph to avoid these operations, DynamiGraph embeds this optimization into its fundamental execution model. Instead of viewing SpDMM as a matrix operation, we treat it as a direct implementation of the message-passing paradigm. The hardware is not programmed to multiply matrices; it is programmed to process a list of edges.

The execution flow is inherently edge-centric. The PE fetches one edge (u,v) with weight $w_{uv}$ at a time. It then performs the necessary data-fetching and computation exclusively for this single connection: it fetches the feature vector for the source node $h_{u}$, computes the message ($w_{uv}\cdot h_{u}$), and aggregates it at the destination node $h_{v}$. In this model, there is no “zero” to be skipped—the hardware only ever performs work where an edge explicitly exists. This guarantees that the computational workload for the SpDMM kernel is always proportional to |E|, never ${\left|V\right|}^{2}$, ensuring maximum efficiency for sparse graphs without requiring any complex static analysis.

#### 5.3.2. Hardware Support for Dynamic Execution

Translating the principle of runtime sparsity exploitation into practice requires specific hardware support within the PE’s control and data paths. We have designed these mechanisms to be both efficient and lightweight, ensuring they do not introduce significant overhead. The key hardware features are as follows:

- Edge-List Data Format. The hardware is built to natively consume graph connectivity data in an edge-list format, specifically a Coordinate-style (COO) list of (source_id, dest_id, weight)tuples. The on-chip Edge Buffer is structured to stream these tuples sequentially to the execution pipeline, which forms the basis of our edge-centric processing model.
- Dedicated SpDMM Instruction. We introduce a specialized high-level instruction, EXEC_SpDMM_RUNTIME. Unlike a generic matrix multiplication command, this instruction explicitly tells the PE to initiate the edge-processing loop. Its operands specify the base addresses of the edge list and feature matrices in the local buffers, along with the total number of edges to be processed in the current task.
- Microcode-driven Control. Upon receiving the EXEC_SpDMM_RUNTIME instruction, the PE’s Instruction Decoder activates a dedicated microcode sequencer. This sequencer implements a hardware loop that orchestrates the entire SpDMM operation on an edge-by-edge basis. For each cycle, it fetches the next edge tuple, issues the appropriate read commands to the Feature Buffer using the source node’s index, and directs the SCK to perform the required scatter-gather arithmetic.
- Fast Indexed Memory Access. A critical prerequisite for this model is the ability to perform fast, indexed lookups into the Feature Buffer. Given a source node index *u* from an edge tuple, the hardware must be able to retrieve the corresponding feature vector $h_{u}$ with minimal latency. We achieve this by implementing the Feature Buffer with banked SRAMs, which allows for parallelized access and helps to hide the latency of data fetching.

#### 5.3.3. SpDMM Execution with Runtime Optimization

The principles and hardware support for our runtime optimization are brought together in the SpDMM execution flow, which is formally described in Algorithm 1. This algorithm outlines the sequence of operations performed within a single PE upon receiving an EXEC_SpDMM_RUNTIME instruction.

The process is fundamentally edge-centric. After initializing an output buffer, the PE’s microcode controller enters a loop that iterates through the assigned partition of the graph’s edge list. In each iteration, it fetches a single edge tuple. The source index is used to retrieve the corresponding feature vector, which is then scaled by the edge weight within the SCK to form a message. This message is immediately accumulated into the register holding the data for the destination node. This tight, hardware-managed loop ensures that computation and data movement are minimal and directly proportional to the number of edges, thereby fully realizing the benefits of runtime sparsity exploitation.

### 5.4. Instruction Set and Compilation Flow

The programmability of DynamiGraph is enabled by a well-defined Instruction Set Architecture (ISA) and a corresponding lightweight compilation flow. This software-hardware interface abstracts the underlying hardware complexity, allowing GNN models to be executed without requiring any hardware reconfiguration.
**Algorithm 1** SpDMM execution incorporating runtime sparsity exploitation.**Require:** Edge list for the partition, $E_{part}=\left\{\left(u,v,w\right),...\right\}$ **Require:** Input feature matrix tile, $H_{in}$ **Require:** Number of edges in the partition, $N_{edges}$ **Ensure:** Output (aggregated) feature matrix tile, $H_{out}$1:Initialize local accumulator buffer $H_{out}$ with zeros.2:**for** i←1 to $N_{edges}$ **do**                              ▷ Loop managed by PE’s microcode controller3:    (u,v,w)← Fetch next edge from on-chip Edge Buffer4:    $h_{u}\leftarrow \mathrm{Read}\;H_{in}\left[u\right]$ from on-chip Feature Buffer                        ▷ Fast indexed access5:    $m\leftarrow w\times h_{u}$                                                         ▷ **Scatter Phase:** Executed in the SCK6:    $H_{out}\left[v\right]\leftarrow H_{out}\left[v\right]+m$                                       ▷ **Gather Phase:** Executed in the SCK7:**end for**8:**return** $H_{out}$


#### 5.4.1. Instruction Set Architecture (ISA)

The DynamiGraph ISA is a specialized set of high-level instructions designed to efficiently control the execution of GCN-like models. Compared to a general-purpose GNN accelerator, our ISA is more streamlined, focusing exclusively on the memory operations and the two computational kernels (GEMM and SpDMM) that our hardware supports. This specialization simplifies the instruction decoder and control logic within the PE.

Each instruction has a uniform length, containing an opcode that defines the operation type, along with fields specifying operands such as memory addresses, data dimensions, and buffer IDs. The key instructions are summarized in Table 8. The most critical instruction is EXEC_SpDMM_RUNTIME, which directly invokes the hardware-managed, edge-centric processing loop that forms the core of our runtime optimization strategy.

#### 5.4.2. Lightweight Compilation

The final component of our software-hardware ecosystem is the compilation flow. In keeping with our philosophy of specialization and efficiency, the compiler for DynamiGraph is intentionally lightweight. It forgoes the complex and time-consuming optimization passes required by more general-purpose GNN accelerators.

General-purpose frameworks often need to perform sophisticated analyses like computation order optimization to handle the diverse layer structures found in models like GAT. In contrast, since DynamiGraph targets a well-defined class of models (e.g., GCN, GraphSAGE) with a predictable layer structure, our compiler’s role is significantly simplified:

1. Template-Based Mapping: The compiler’s primary function is to parse the input GNN model and perform a direct, template-based mapping of its layers to our ISA. A standard GCN layer, for instance, is always translated into a fixed sequence of instructions: MEM_LOAD to fetch data, EXEC_SpDMM_RUNTIME for aggregation, EXEC_GEMM for transformation, and MEM_STORE to write back the results.
2. Data Preparation: The compiler’s other main responsibility is to prepare the data for the hardware. It partitions the graph’s node features and edge list into tiles that fit within the PEs’ local on-chip buffers and ensures the graph connectivity is provided in the hardware-native COO edge-list format.

This streamlined, template-based approach makes the compilation process exceptionally fast, typically completing in milliseconds. This ensures that the host-side software overhead is minimal, which is critical for achieving low end-to-end inference latency and a key advantage of the DynamiGraph system.

### 5.5. Theoretical Latency Model

The total inference latency is primarily composed of the time spent on computation and the time spent on data movement. We first model the execution latency of the core computational kernels, assuming all necessary data has been loaded into the PE’s local on-chip buffers.

#### 5.5.1. Latency of Computational Kernels

The execution time for each GNN operation is determined by the total number of floating-point operations (FLOPs) and the throughput of the Specialized Computation Kernel (SCK) in its corresponding mode.

**Theorem** **1.** *The execution latency of the GEMM kernel ($T_{GEMM}$) for an input feature matrix $H\in R^{N\times D_{in}}$ and weight matrix $W\in R^{D_{in}\times D_{out}}$ on a single PE with a computational throughput of $P_{GEMM}$ FLOPS is given by:*

$$T_{GEMM}=\frac{2\cdot N\cdot D_{in}\cdot D_{out}}{P_{GEMM}}$$

*Thus, the latency is directly proportional to the product of the matrix dimensions, i.e., $T_{GEMM}\propto N\cdot D_{in}\cdot D_{out}$.*

**Proof.**  The dense matrix multiplication H×W requires $N\cdot D_{in}\cdot D_{out}$ multiply-accumulate (MAC) operations. Since each MAC consists of one multiplication and one addition, the total number of FLOPs is $2\cdot N\cdot D_{in}\cdot D_{out}$. Given that the SCK in GEMM mode can sustain a throughput of $P_{GEMM}$ floating-point operations per second, the total time required is the total number of FLOPs divided by the throughput. □

**Theorem** **2.** *The execution latency of the SpDMM kernel ($T_{SpDMM}$), leveraging runtime sparsity exploitation for a graph with |E| edges and a feature dimension of $D_{in}$, on a single PE with a computational throughput of $P_{SpDMM}$ FLOPS is given by:*

$$T_{SpDMM}=\frac{2\cdot \left|E\right|\cdot D_{in}}{P_{SpDMM}}$$

*Thus, the latency is directly proportional to the product of the number of edges and the feature dimension, i.e., $T_{SpDMM}\propto \left|E\right|\cdot D_{in}$.*

**Proof.**  Due to the edge-centric execution model of DynamiGraph, computation is only performed for non-zero edges. For each of the |E| edges, the operation consists of scaling a source feature vector of length $D_{in}$ by the edge weight and accumulating the result. This vector operation requires $D_{in}$ multiplications and $D_{in}$ additions, for a total of $2\cdot D_{in}$ FLOPs per edge. The total computational workload is therefore $2\cdot \left|E\right|\cdot D_{in}$ FLOPs. With a sustained throughput of $P_{SpDMM}$ FLOPS for the SCK in SpDMM mode, the execution time is the total FLOPs divided by the throughput. □

#### 5.5.2. End-to-End Inference Latency

Building upon the kernel-level models, we now formulate a model for the total end-to-end inference latency ($T_{E2E}$). This model accounts not only for the computation time but also for the overhead of data movement from off-chip memory and the performance gains from parallel execution across multiple PEs.

**Theorem** **3.** *The end-to-end inference latency ($T_{E2E}$) for an L-layer GCN, executed on DynamiGraph with $N_{PE}$ processing elements and an effective memory bandwidth of B, can be modeled as the sum of the latencies for each layer:*

$$T_{E2E}\approx \sum_{l=1}^{L}\left(T_{transfer}^{\left(l\right)}+\frac{T_{SpDMM}^{\left(l\right)}+T_{GEMM}^{\left(l\right)}}{N_{PE}}\right)$$

*where $T_{transfer}^{\left(l\right)}$ is the data transfer time for layer l, and $T_{SpDMM}^{\left(l\right)}$ and $T_{GEMM}^{\left(l\right)}$ are the total computational latencies for the kernels at that layer, as defined in Theorems 1 and 2.*

**Proof.**  The proof follows from analyzing the execution of a single GNN layer and summing across all layers.

1. Layer-by-Layer Execution: A GNN is executed sequentially, one layer at a time. Therefore, the total latency is the summation of the latencies of each of the *L* layers.
2. Single-Layer Latency ($T_{layer}^{\left(l\right)}$): The time to process a single layer *l* consists of two primary phases: data transfer and computation.
  - Data Transfer ($T_{transfer}^{\left(l\right)}$): Before computation, the input data for the layer (node features and graph structure) must be loaded from the off-chip DDR to the on-chip buffers of the PEs. The time for this is $T_{transfer}^{\left(l\right)}={\mathrm{DataSize}}^{\left(l\right)}/B$. While techniques like double-buffering are used to hide this latency by overlapping it with computation from the previous layer, it remains a fundamental component of the time budget.
  - Parallel Computation: The total computational work for a GCN layer ($T_{compute}^{\left(l\right)}=T_{SpDMM}^{\left(l\right)}+T_{GEMM}^{\left(l\right)}$) is partitioned and distributed across the $N_{PE}$ available processing elements. Assuming ideal load balancing by the Task Scheduler, the parallel execution time is the total computation time divided by the number of PEs.
3. Total Latency: Combining these components, the latency for a single layer is approximately $T_{layer}^{\left(l\right)}=T_{transfer}^{\left(l\right)}+T_{compute}^{\left(l\right)}/N_{PE}$. Summing this across all *L* layers yields the final expression for the total end-to-end latency, $T_{E2E}$. This model provides a comprehensive framework for predicting performance and identifying potential bottlenecks in the system.

□

### 5.6. Experimental Setup

#### 5.6.1. Datasets

To comprehensively evaluate the performance of our proposed DynamiGraph accelerator, we utilize a suite of widely-recognized benchmark graph datasets. These datasets were chosen to cover a broad spectrum of scales and characteristics, ranging from small citation networks to large-scale social networks and product graphs. This variety allows us to rigorously test the scalability and efficiency of DynamiGraph, particularly its core runtime sparsity exploitation mechanism. The key statistics of these datasets, including the number of vertices (|V|), edges (|E|), the dimension of node features, and the number of classes, are summarized in Table 9.

#### 5.6.2. Evaluation Platform

All experiments were conducted to evaluate the performance of DynamiGraph on a single, resource-constrained FPGA platform, representative of an edge computing device. This approach allows for a focused analysis of the architecture’s efficiency and scalability in non-datacenter environments.

The key specifications of our evaluation platform are detailed in Table 10. The selected device features significantly fewer logic and memory resources compared to high-end accelerator cards, providing a realistic testbed for our evaluation.

#### 5.6.3. Evaluation Metrics

To conduct a quantitative and multi-faceted evaluation of DynamiGraph, we employ a set of well-defined metrics. These are chosen to assess the raw performance, the effectiveness of the core architectural optimizations, and the implementation cost on our target hardware.

- End-to-End Latency. This is the primary performance metric, measured in milliseconds (ms). It is defined as the total time elapsed from the moment the GNN model and graph data are supplied to the accelerator to the moment the final node embeddings are produced.
- Throughput. Measured in inferences per second (inferences/s), throughput is the reciprocal of latency. It indicates the system’s capacity for serving continuous inference requests, a crucial factor for deployment.
- FLOPs Reduction. This metric directly quantifies the effectiveness of our core runtime sparsity exploitation. We measure the actual number of floating-point operations (FLOPs) executed by the SpDMM kernel and compare it to the theoretical FLOPs of an equivalent dense matrix multiplication. This highlights the computational work saved on sparse graphs.
- FPGA Resource Utilization. We report the estimated utilization of key FPGA resources, including Look-Up Tables (LUTs), Digital Signal Processors (DSPs), and Block RAM (BRAMs). This metric is essential for evaluating the hardware implementation cost and viability of our design on the resource-constrained target platform.

## Figures and Tables

**Table 1:** Speedup achieved by increasing the number of PEs relative to the 1-PE baseline (Reddit dataset, Hidden Dim = 128).

PEs	Latency (ms)	Speedup vs. 1 PE
1	170.01	1.00×
2	90.12	1.89×
4	50.05	3.40×
8	27.21	6.25×

**Table 2:** Analytical hardware resource estimation of the 8-PE DynamiGraph architecture on the AXU2CGA edge platform (Chip: XCZU2CG, Target Frequency: 150 MHz).

Resource Type	Available (XCZU2CG)	Estimated Used	Utilization (%)
Look-Up Tables (LUT)	47,200	21,860	46.31%
Flip-Flops (FF)	94,400	32,790	34.74%
DSP Slices	240	128	53.33%
Block RAM (BRAM, Mb)	5.3	2.5	47.17%
UltraRAM (URAM, Mb)	0.0	0.0	0.00%

**Table 3:** Analytical power consumption profile and peak efficiency projections on the AXU2CGA platform.

Parameter/Performance Metric	Value
Evaluation Methodology	Pre-Route Analytical Modeling
Target Frequency (MHz)	150
Core Supply Voltage (V)	0.85
Dynamic Power Projection (W)	1.85
Static Power Baseline (W)	0.65
Total Estimated On-Chip Power (W)	2.50
Peak Computing Throughput (GOPs)	38.40
Energy Efficiency Ratio (GOPs/W)	15.36

**Table 4:** Performance and energy efficiency comparison against the sparse software framework (PyTorch Geometric) on the representative edge CPU platform.

Platform	Latency (ms)			Energy per Inference (mJ)		
	Cora	PubMed	Reddit	Cora	PubMed	Reddit
Edge CPU Baseline (PyG)	10.26	49.25	-	35.89	172.37	-
DynamiGraph (Ours)	0.68	2.65	27.21	1.70	6.63	68.03

**Table 5:** Normalized architectural and resource efficiency comparison with state-of-the-art FPGA GNN accelerators.

Metric	AWB-GCN	GraphAGILE	DynamiGraph (Ours)
Target Platform	Stratix 10 (Datacenter)	Alveo U200 (Datacenter)	AXU2CGA (Edge)
Supported Kernels	GEMM, SpDMM	GEMM, SpDMM, SDDMM	GEMM, SpDMM
Sparsity Handling	Dynamic Workload Bal.	Static Compilation	Runtime Edge-Centric
Peak Throughput (GOPs)	3584.0	1459.2	38.40
DSP Slices Used	3960	4096	128
Norm. DSP Efficiency (GOPs/DSP)	0.90	0.35	0.60

**Table 6:** Feature Buffer access statistics and on-chip cache hit rates across sub-graphs of varying densities (Simulated on 10,000 nodes, 1000-node Cache Capacity).

Average Node Degree	Total Feature Accesses	Off-Chip DDR4 Reads	On-Chip Cache Hit Rate (%)
10	49,975	7172	85.65%
20	99,900	8325	91.67%
50	249,375	9243	96.29%
100	497,500	9610	98.07%

**Table 7:** Comparison of DynamiGraph with state-of-the-art GNN accelerators.

Accelerator	Platform	Target Scenario	Supported Kernels	Sparsity Optimization	Architecture Type
HyGCN	ASIC	General Purpose	GEMM, SpDMM	Static	Fixed Architecture
AWB-GCN	FPGA (Stratix 10)	Cloud/Datacenter	GEMM, SpDMM	Dynamic Workload Balancing	Fixed Architecture
GraphAGILE	FPGA (Alveo U200)	Cloud/Datacenter	GEMM, SpDMM, SDDMM	Static	General Overlay
DynamiGraph (Ours)	FPGA (AXU2CGA)	Edge/IoT	GEMM, SpDMM	Runtime Edge-Centric	Specialized Overlay

**Table 8:** Key high-level instructions supported by the DynamiGraph ISA.

Instruction	Opcode	Description
MEM_LOAD/STORE	0 × 01/0 × 02	Initiates a DMA transfer of a data block (features, edges, or weights) between off-chip DDR and a PE’s local on-chip buffer.
EXEC_GEMM	0×10	Configures the SCK to operate in GEMM (systolic array) mode and executes a dense matrix multiplication on data in the local buffers.
EXEC_SpDMM_RUNTIME	0×11	Configures the SCK for SpDMM and initiates the edge-centric processing loop, which dynamically exploits graph sparsity.

**Table 9:** Statistics of the benchmark graph datasets used in our evaluation.

Dataset	Vertices (|V|)	Edges (|E|)	Features	Classes
Citeseer (CI)	3327	4732	3703	6
Cora (CO)	2708	5429	1433	7
PubMed (PU)	19,717	44,338	500	3
Flickr (FL)	89,250	899,756	500	7
Reddit (RE)	232,965	11,606,919	602	41
Yelp (YE)	716,847	6,977,410	300	100
Amazon-Products (AP)	1,569,960	264,339,468	200	107

**Table 10:** Specifications of the DynamiGraph evaluation platform.

Parameter	Specification
FPGA Device	AXU2CGA Edge Board (Xilinx XCZU2CG)
Logic Resources (LUTs)	~47 K
DSP Slices	240
On-Chip Memory	~2.5 MB (BRAM)
Target Frequency	150 MHz
Off-Chip Memory	2 GB DDR4

## Data Availability

The datasets analyzed during the current study are available from the corresponding author on reasonable request.
